# Autochthonous Dengue Outbreak, Paris Region, France, September–October 2023

**DOI:** 10.3201/eid2912.231472

**Published:** 2023-12

**Authors:** Marta Zatta, Ségolène Brichler, William Vindrios, Giovanna Melica, Sébastien Gallien

**Affiliations:** Henri Mondor University Hospital, Assistance Publique-Hôpitaux de Paris, Paris, France (M. Zatta, W. Vindrios, G. Melica, S. Gallien);; Avicenne University Hospital, Assistance Publique-Hôpitaux de Paris, Paris, France (S. Brichler)

**Keywords:** Dengue, viruses, arbovirus, vector-borne infections, zoonoses, mosquitoes, Aedes albopictus, France, Paris, Europe

## Abstract

We describe clinical and laboratory findings of 3 autochthonous cases of dengue in the Paris Region, France, during September–October 2023. Increasing trends in cases, global warming, and growth of international travel mean that such infections likely will increase during warm seasons in France, requiring stronger arbovirus surveillance networks.

In Europe, dengue primarily is imported from endemic countries. However, an increasing number of autochthonous cases and limited outbreaks have been described since 2010. In 2023, a total of 105 autochthonous cases were reported: 66 in Italy, 36 in France; and 3 in Spain (*1*). We describe an autochthonous outbreak of dengue in the Paris Region of France during September–October 2023.

## The Study

On September 13, 2023, a 36-year-old woman from Limeil-Brévannes, a city 15 km south of Paris, who had no consistent medical history started having symptoms of fever (>38°C), malaise, and headache. Her 7-year-old son experienced the same symptoms during September 11–13 and had a maculopapular rash that disappeared 5 days after the onset. Also, the woman’s partner had symptoms begin on September 14 and had a fever (>38°C), chills, frontal headache, myalgia, a papular rash, and itching on the trunk and upper limbs. The case-patients reported no travel abroad or to other regions of France.

On September 19, the woman continued to be feverish daily and began to have nausea and vomiting. She consulted the emergency department of Hôpital Henri Mondor, Créteil, south of Paris, where blood tests were performed. Tests revealed thrombocytopenia (105,000 cells/μL; reference range 150,000–450,000 cells/μL), leukopenia (700/μL; reference range 1,000–4,000 U/L), and increased alanine aminotransferase (213 U/L; reference range <35 U/L), aspartate aminotransferase (266 U/L; reference range <35 U/L), and gamma-glutamyl transferase (135 U/L; reference range <40 U/L). C-reactive protein was 2.4 mg/L (reference range <5 mg/L). Major and common causes of viral hepatitis were excluded, and serologic tests for hepatitis A–C, HIV, and cytomegalovirus were negatives. Epstein-Barr virus serology was compatible with a previous infection.

Because a diagnosis was not made during clinical examination, we performed arbovirus serologic testing, including for dengue virus (DENV), on September 22. DENV serologic results were IgM positive; thus, we performed a retrospective analysis of blood samples collected on September 19. In that analysis, results for DENV nonstructural protein 1 (NS1) antigen in plasma were positive, as was the real-time reverse transcription PCR (RT-PCR), which could identify DENV type 2 (cycle threshold 35), confirming the diagnosis of acute DENV infection.

We performed a second serologic test 25 days after symptom onset, which showed seroconversion for DENV, IgM persistence, and apparition of IgG (Table). The patient’s son and partner also had DENV serologic testing on October 13, and results were IgM and IgG positive. All 3 patients fully recovered, and no case was classified as severe dengue.

**Table:** Clinical and laboratory findings in an outbreak of autochthonous dengue, Paris Region, France, September–October 2023*

Clinical, epidemiologic and laboratory parameters	Case-patient 1			Case-patient 2	Case-patient 3
Date of symptom onset	Sep 13			Sep 11	Sep 14
Symptoms	Fever, malaise, frontal headache, nausea and vomiting			Fever, malaise, frontal headache, and maculopapular rash	Fever, chills, frontal headache, myalgia, papular rash, and trunk and upper limbs itching
Date of symptom resolution	Sep 21			Sep 14	Sep 21
Epidemiologic link	Index case			Household contact of index case	Household contact of index case
Sample collection dates	Sep 19	Sep 22	Oct 7	Oct 13	Oct 13
Delay between sample collection and symptom onset, d	7	10	25	33	30
DENV RNA in blood	DENV-2†	Not tested	Not tested	Not tested	Not tested
DENV NS1 antigen	Positive‡	Not tested	Not tested	Not tested	Not tested
DENV IgM	Not tested	Positive.§ r = 72	Positive.§ r = 70	Positive,¶ r = 30	Positive,¶ r = 28
DENV IgG	Not tested	Negative.§ r<5	Positive.§ r = 12	Positive,¶ r = 15	Positive,¶ r = 17

*DENV, dengue virus; NS1, nonstructural protein 1; r, ratio; RT-PCR, reverse transcription PCR. †DENV RNA was amplified, after purifying total nucleic acids from plasma, by real-time RT-PCR by using Realstar Dengue RT-PCR kit 3.0 and RealStar Dengue Type RT-PCR Kit 1.0 (Altona Diagnostics, https://www.altona-diagnostics.com). ‡DENV NS1 antigen was detected in plasma by using Dengue NS1 Ag Strips (Biosynex SA, https://www.biosynex.com) immunochromatographic assay. §DENV IgM and IgG were detected by using Virclia Dengue IgM/IgG (Orgentec, https://www.orgentec.com/en) chemiluminescence immunoassay; results are positive if r>11. ¶DENV IgM and IgG were detected by Vircell Dengue IgM/IgG (Orgentec) ELISA immunoassay; results are positive if r>11.

According to national guidelines, we made a notification to the national health authority of France on October 16, which prompted a door-to-door survey in the family’s neighborhood during October 19–20. Pesticide spraying for mosquito control was also performed in the neighborhood.

In Europe, since 2010, autochthonous dengue cases have been reported in several countries (*1**–**3*). In France, a case was reported in Nice in 2010, after which the number of case reports has been increasing, and 9 events of autochthonous DENV transmission were identified in 2022, resulting in 65 autochthonous cases (*3*,*4*). To date, all autochthonous dengue cases occurred in the south of France, and none had been described in the Paris Region.

In the reported family cluster, the virus was probably introduced into mainland France through viremic travelers returning from an endemic area. Indeed, among countries that reported a considerable increase in dengue cases in recent years, some are overseas territories of France (*5*,*6*). In an ongoing 2023 outbreak in Martinique and Guadeloupe, DENV-2 serotype has been identified in most cases (*7*). The cluster we report was caused by DENV-2, but past autochthonous DENV transmission events in France were caused by DENV-1 and DENV-3 (*4*). Clinicians should consider the serotype of DENV because infection with any DENV serotype will cause an adaptive immune response to develop, which provides short-term immunity against heterologous DENV serotypes (*8*,*9*). In addition, priming with 1 DENV serotype can increase the risk for severe dengue upon future infection with a heterologous virus (*8*,*9*).

Dengue is transmitted by the bite of an infected female mosquito. The *Aedes aegypti* mosquito, the primary vector, has not been established in continental Europe (*10*,*11*). *Ae. albopictus*, a less competent vector, might act as an epidemic driver in areas where *Ae. aegypti* is absent (*10*,*11*). Since the 1990s, *Ae. albopictus* has increasingly been detected in Europe and France. In 2022, *Ae. albopictus* was found in 71 of 95 French departments, and since 2015, it has been found in the area where the described family lives (*12*).

Two main elements might contribute to an increasing risk for autochthonous arbovirus transmission: intensification in international travel observed in recent decades and more stable vector mosquito populations outside previously known endemic areas. International travel raises arbovirus importation in nonendemic countries, and global warming could contribute to establishment of a more stable vector population, such as *Ae. albopictus*, in France. Those factors underline several points from this case report.

Like most viral infections, dengue has a large spectrum of clinical manifestations, as seen in the cases we report. Most persons remain asymptomatic or develop minor symptoms, but ≈25% experience a self-limited febrile illness, accompanied by mild-to-moderate hematologic and biochemical abnormalities (*13*). Faced with compatible clinical cases without other confirmed diagnoses, clinicians in nonendemic areas should also test for an arbovirus infection during summer and autumn. Case detection is crucial for implementing the necessary public health measures to prevent further virus transmission.

The choice of laboratory test depends on the number of days after illness onset. Before day 7, dengue can be diagnosed by detection of viral RNA or antigens (*13*). DENV IgM starts to rise from day 4, peak around days 10–14, and then decline and disappear after ≈3 months. In primary infections, dengue IgG can be detected at low concentrations by the end of the second week; the concentration increases slowly thereafter and is thought to persist for life (*13*). Often, paired acute and convalescent samples are required if direct detection of the virus is not available. Diagnosis can be accelerated when clinicians make every effort to obtain RT-PCR or antigenic testing, including reanalyzing samples taken in the viremic phase, when possible, which enables diagnosis without waiting for seroconversion.

## Conclusions

In this report, we described a cluster of autochthonous dengue in the Paris Region of France. We assume that this cluster might be only the tip of the iceberg of a wider latent DENV reservoir in the Paris area due to the stable presence of the vector coexisting with viremic persons returning from endemic countries. We believe that paying close attention to the autochthonous transmission of arboviruses is crucial because recent increasing trends in cases, global warming, and the growth of international travel very likely mean that such infections will increase during warm seasons in France. Because of the short stay, implications for tourists visiting Paris are negligible and public health authorities do not recommend specific prophylactic strategies (*14*). However, for the 2024 Summer Olympic Games, when millions of visitors are expected in Paris, increased attention to tracking arboviruses, including dengue virus, is advisable.

## References

[1] European Centre for Disease Prevention and Control. Autochthonous vectorial transmission of dengue virus in mainland EU/EEA, 2010–present [cited 2023 Oct 31]. https://www.ecdc.europa.eu/en/all-topics-z/dengue/surveillance-and-disease-data/autochthonous-transmission-dengue-virus-eueea

[2] Lazzarini L, Barzon L, Foglia F, Manfrin V, Pacenti M, Pavan G, et al. First autochthonous dengue outbreak in Italy, August 2020. Euro Surveill. 2020;25:2001606. 10.2807/1560-7917.ES.2020.25.36.2001606 32914745 PMC7502902

[3] La Ruche G, Souarès Y, Armengaud A, Peloux-Petiot F, Delaunay P, Desprès P, et al. First two autochthonous dengue virus infections in metropolitan France, September 2010. Euro Surveill. 2010;15:19676. 10.2807/ese.15.39.19676-en 20929659

[4] Cochet A, Calba C, Jourdain F, Grard G, Durand GA, Guinard A, et al. Investigation team. Autochthonous dengue in mainland France, 2022: geographical extension and incidence increase. Euro Surveill. 2022;27:44. 10.2807/1560-7917.ES.2022.27.44.2200818 PMC9635021 36330819

[5] European Centre for Disease Prevention and Control. Local transmission of dengue fever in France and Spain–2018 [cited 2023 Oct 31]. https://www.ecdc.europa.eu/sites/default/files/documents/08-10-2018-RRA-Dengue-France.pdf

[6] Santé publique France. Dengue fever in the Antilles. Update as of October 5, 2023 [in French] [cited 2023 Oct 20]. https://www.santepubliquefrance.fr/regions/antilles/documents/bulletin-regional/2023/dengue-aux-antilles.-point-au-5-octobre-2023

[7] Santé publique France. The dengue epidemic declared in Martinique and Guadeloupe: protect yourself! [in French] [cited 2023 Oct 20]. https://www.santepubliquefrance.fr/les-actualites/2023/l-epidemie-de-dengue-declaree-en-martinique-et-en-guadeloupe-protegez-vous

[8] Wilder-Smith A, Ooi EE, Horstick O, Wills B. Dengue. Lancet. 2019;393:350–63. 10.1016/S0140-6736(18)32560-1 30696575

[9] Guzman MG, Halstead SB, Artsob H, Buchy P, Farrar J, Gubler DJ, et al. Dengue: a continuing global threat. Nat Rev Microbiol. 2010;8(Suppl):S7–16. 10.1038/nrmicro2460 21079655 PMC4333201

[10] Lambrechts L, Scott TW, Gubler DJ. Consequences of the expanding global distribution of *Aedes albopictus* for dengue virus transmission. PLoS Negl Trop Dis. 2010;4:e646. 10.1371/journal.pntd.0000646 20520794 PMC2876112

[11] Messina JP, Brady OJ, Scott TW, Zou C, Pigott DM, Duda KA, et al. Global spread of dengue virus types: mapping the 70 year history. Trends Microbiol. 2014;22:138–46. 10.1016/j.tim.2013.12.011 24468533 PMC3946041

[12] Institut Pasteur. Tiger mosquito in France: 71 departments on red alert [cited 2023 Oct 18]. https://www.pasteur.fr/en/research-journal/news/tiger-mosquito-france-71-departements-red-alert

[13] World Health Organization. Dengue guidelines for diagnosis, treatment, prevention and control: new edition. Geneva: The Organization; 2009. 23762963

[14] Centers for Disease Control and Prevention. Dengue: plan for travel [cited 2023 Oct 31]. https://www.cdc.gov/dengue/prevention/plan-for-travel.html

